# Supplementary material for: Metabolic Engineering of Pichia pastoris for the Production of Triacetic Acid Lactone
Source: J Fungi (Basel). 2023 Apr 20;9(4):494. doi: 10.3390/jof9040494 (PMC10145311; doi:10.3390/jof9040494)
Supplement: Supplementary file 1 [file jof-09-00494-s001.zip › jof-2269706-supplementary.pdf]

Supplementary Figures

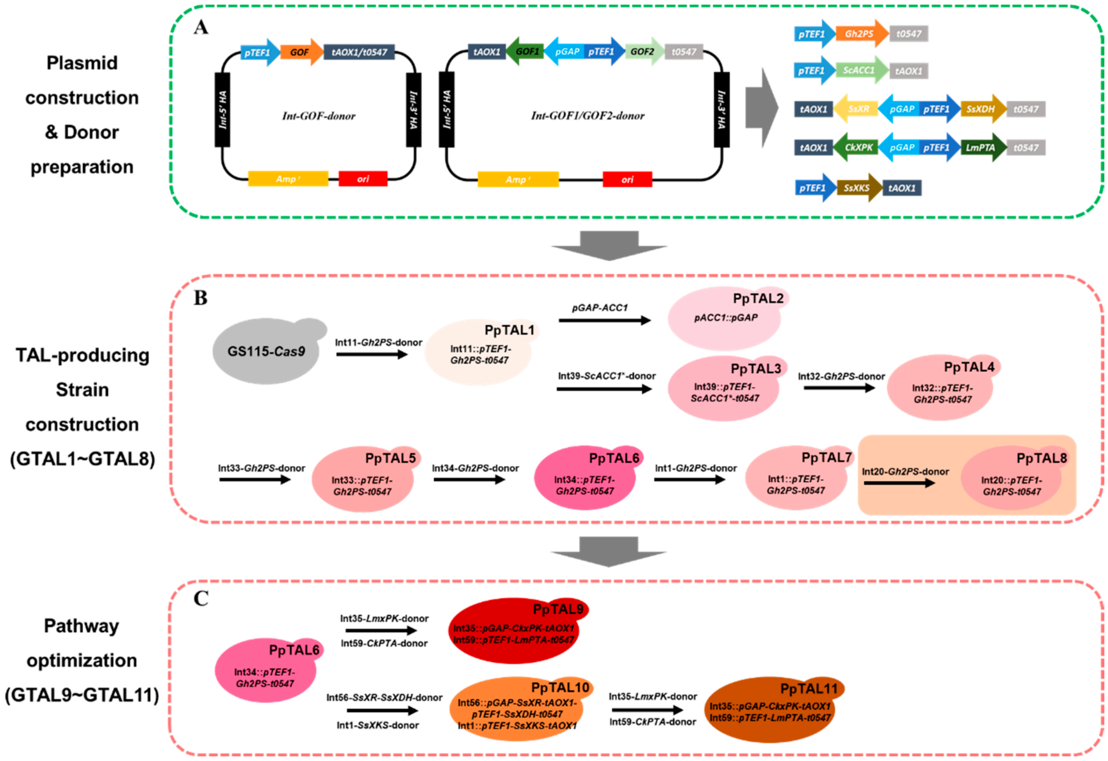

Figure S1. Schematic diagram of plasmid and strain construction in this study. (A) Schematic diagram of gene integration expression cassette and plasmid construction. (B) Schematic diagram of TAL-producing strains construction. (C) Schematic diagram of the pathway optimization strain construction for enhanced acetyl-CoA synthesis and/or xylose utilization.

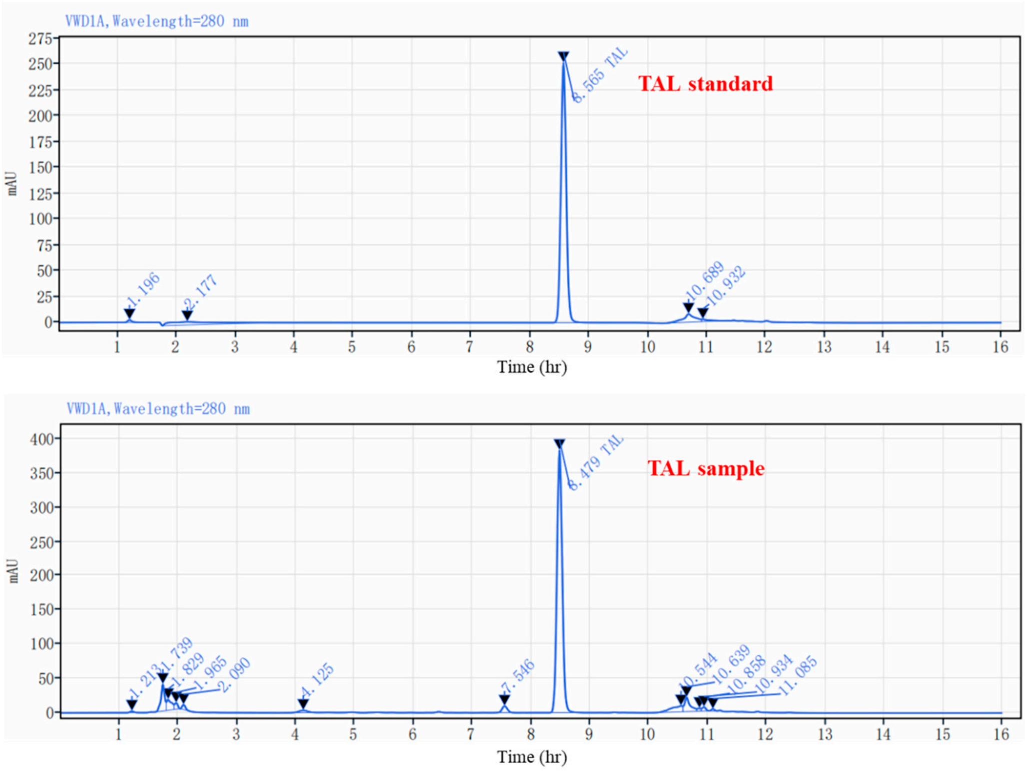

Figure S2. HPLC analysis for the identification and quantification of TAL. The peak corresponding to TAL was observed at ~8.50 min.

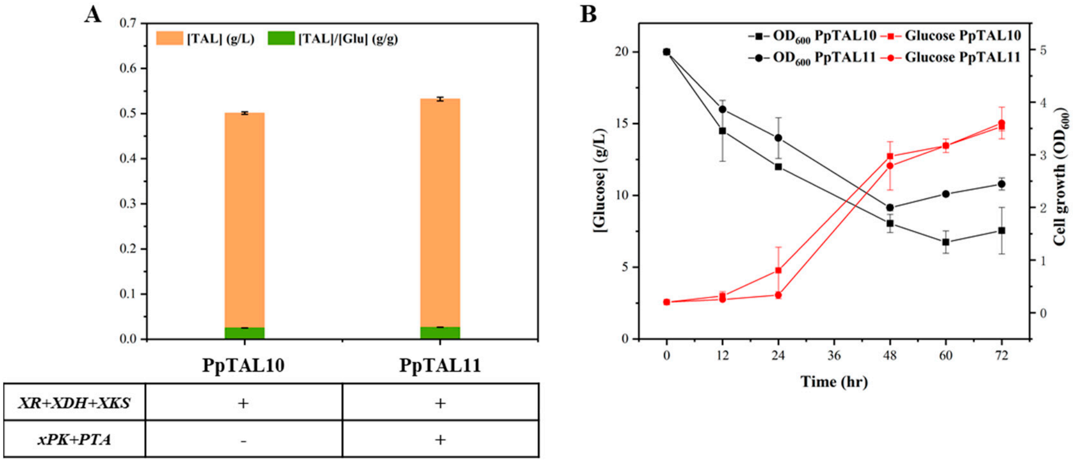

Figure S3. TAL production from glucose. (A) Comparison of TAL titer of the xylose utilization strain with or without the PK pathway in SCD medium. (B) Glucose consumption and cell growth profiles of PpTAL10 and PpTAL11 in SCD medium. The data represent three biological replicates and the error bars represent standard deviations.

Supplementary Tables

Table S1. List of Plasmids Used in This Study.

Name	Genotype	Description
HZP-gRNA-Int1	PARS1, zeocin, *pSER*, Int1	The sgRNA helper plasmids
HZP-gRNA-Int11	PARS1, zeocin, *pSER*, Int11	
HZP-gRNA-Int20	PARS1, zeocin, *pSER*, Int20	
HZP-gRNA-Int32	PARS1, zeocin, *pSER*, Int32	
HZP-gRNA-Int33	PARS1, zeocin, *pSER*, Int33	
HZP-gRNA-Int34	PARS1, zeocin, *pSER*, Int34	
HZP-gRNA-Int35	PARS1, hygromycin B, *pSER*, Int35	
HZP-gRNA-Int39	PARS1, zeocin, *pSER*, Int39	
HZP-gRNA-Int56	PARS1, zeocin, *pSER*, Int56	
HZP-gRNA-Int59	PARS1, zeocin, *pSER*, Int59	
HZP-gRNA-Det*PFK1*	PARS1, zeocin, *pSER*, Det*PFK1*	
HZP-gRNA-Det*PYK1*	PARS1, zeocin, *pSER*, Det*PYK1*	
Int1-donor	Amp, Int1-*pTEF1*-*Hind*III-*Nde* I -*t0547*	The integration donor helper plasmids containing an upstream and a down-stream homologous arm of ~500 bp as well as the restriction sites for genes or genes cassettes
Int11-donor	Amp, Int11-*pTEF1*-*Hind*III-*Nde* I -*t0547*	
Int20-donor	Amp, Int20-*pTEF1*-*Hind*III-*Nde* I -*t0547*	
Int32-donor	Amp, Int32-*pTEF1*-*Hind*III-*Nde* I -*t0547*	
Int33-donor	Amp, Int33-*pTEF1*-*Hind*III-*Nde* I -*t0547*	
Int34-donor	Amp, Int34-*pTEF1*-*Hind*III-*Nde* I -*t0547*	
Int35-donor	Amp, Int35-*pGAP*-*Ava* I -*Bgl* II -*tAOX1*	
Int39-donor	Amp, Int39-*pTEF1*-*Hind*III-*Nde* I -*tAOX1*	

Int56-donor	Amp, Int56-*pTEF1-Hind*III-*Nde* Ⅰ *-t0547-pGAP-Ava* Ⅰ *-Bgl* Ⅱ *-tAOX1*	
Int1-donor	Amp, Int1-*pTEF1-Hind*III-*Nde* Ⅰ *-tAOX1*	
Int59-donor	Amp, Int59-*Not* Ⅰ *-Nde* Ⅰ	
Det*PFK1*-donor	Amp, Det*PFK1*-HA	The deletion donor helper plasmids containing an upstream and a downstream homologous arm of ~500 bp
Det*PYK1*-donor	Amp, Det*PYK1*-HA	
Int1-*Gh2PS*-donor	Amp, Int1-*pTEF1-Gh2PS-t0547*	Genes cloned into the integration donor helper plasmids for site-specific integration
Int11-*Gh2PS*-donor	Amp, Int11-*pTEF1-Gh2PS-t0547*	
Int20-*Gh2PS*-donor	Amp, Int20-*pTEF1-Gh2PS-t0547*	
Int32-*Gh2PS*-donor	Amp, Int32-*pTEF1-Gh2PS-t0547*	
Int33-*Gh2PS*-donor	Amp, Int33-*pTEF1-Gh2PS-t0547*	
Int34-*Gh2PS*-donor	Amp, Int34-*pTEF1-Gh2PS-t0547*	
Int35-*CkxPK*-donor	Amp, Int35-*pGAP-CkxPK-tAOX1*	
Int39-*ScACC1**-donor	Amp, Int39-*pTEF1-ScACC1*-tAOX1*	
Int56-*SsXR-SsXDH*-donor	Amp, Int56-*pTEF1-SsXDH-t0547-pGAP-SsXR-tAOX1*	
Int1-*SsXKS*-donor	Amp, Int1-*pTEF1-SsXKS-tAOX1*	
Int59-*LmPTA*-donor	Amp, Int59-*pAOX1-LmPTA-tAOX1*	

Table S2. List of Primers Used in This Study.

**Primers**	**Sequences (5′-3′)**
sgRNA-Int1-F[1]	ACGCTATCTGAAGTATTTACTGGG
sgRNA-Int1-R	AAACCCCAGTAAATACTTCAGATA
sgRNA-Int11-F	ACGCTATTAAAAAAGACGATCCCG
sgRNA-Int11-R	AAACCGGGATCGTCTTTTTTAATA
sgRNA-Int20-F	ACGCAGAAGAAAATGCGAAACAGG
sgRNA-Int20-R	AAACCCTGTTTCGCATTTTCTTCT
sgRNA-Int32-F	ACGCGTGACGAAAGAGATGAGGTG
sgRNA-Int32-R	AAACCACCTCATCTCTTTCGTCAC
sgRNA-Int33-F	ACGCCCGTCACTATGAGGACAAAG
sgRNA-Int33-R	AAACCTTTGTCCTCATAGTGACGG
sgRNA-Int34-F	ACGCGATCAGTTCATTGATAGACA
sgRNA-Int34-R	AAACTGTCTATCAATGAACTGATC
sgRNA-Int35-F	ACGCACTGATTTGCCAGAAGAACA
sgRNA-Int35-R	AAACTGTTCTTCTGGCAAATCAGT
sgRNA-Int39-F	ACGCGATTCAGTAGAGTCCTATTG
sgRNA-Int39-R	AAACCAATAGGACTCTACTGAATC
sgRNA-Int56-F	ACGCGAAATGTTGCTCGGTGCGCG
sgRNA-Int56-R	AAACCGCGCACCGAGCAACATTTC
sgRNA-Int59-F	ACGCAATGATGTCAATCCCATACG
sgRNA-Int59-R	AAACCGTATGGGATTGACATCATT
Gh2PS-F	TATTCTCACTACATACATTTAGTTATTCGCCAACGATGG-GAAGTTACTCTAGCGATG
Gh2PS-R	TTAGTTTAGTCTTAAACTAAGCGAAACTACGTACGTCAATTCCCATTTGC-TACAGCTG

*ScACC1**-F	TTTATTCTCACTACATACATTTTAGTTATTCGCCAACATGAGCGAAGAAA-GCTTATTC
*ScACC1**-R	GGCAAATGGCATTCTGACATCCTCTTGATTAGAATCTAG-TCATTTCAAAGTCTTCAAC
SsXR-F	CAATTGAACAACTATCAAAACACACATGCCTTCTATTAAGTTGAACTCTG
SsXR-R	AATGGCATTCTGACATCCTCTTGAATTAGACGAAGATAGGAATCTTGTCC
SsXDH-F	CATACATTTTAGTTATTCGCCAACAATGACTGCTAACCCTTCCTTG
SsXDH-R	TAAACTAAGCGAAACTACGTACGCATTACTCAGGGCCGTCAATGA
SsXKS-F	ACATACATTTTAGTTATTCGCCAACATGACCACTACCCCATTTGA
SsXKS-R	TGACATCCTCTTGATTAGAATCTAGTTAGTGTTTCAATTCACTTTCCATCT
CkxPK-F	TCAATTGAACAACTATCAAAACACACAATGGCTGATTTCGACTCAAA
CkxPK-R	TTGAAGTGAAAAAAAGAAATGCACGACTTATGCAAATTAGCATCTCAC-TTAATCTTCTGT
LmPTA-F	ACATACATTTTAGTTATTCGCCAACGCATGAAGCTAATGGAAAACATCTTC
LmPTA-R	TAAACTAAGCGAAACTACGTACGCTTATTAGCCTTGCGCTTGC
Int1-*Gh2PS*-F	TACTTCTTTTGCAACGTAAATACTAGTCTAAAAGCTCAAGGAGATTTCAGAGGAG-TTG
Int1-*Gh2PS*-R	GCCTTAAAGGAAATTTGGAACTCCAGTGCCACTTTCGCTAGGGTTGTTCCGTTAG-CAC
Int11-*Gh2PS*-F	ATTAAATTTGGCAATTCCTCTAACATGCCATGGGATCAAGGAGATTTCAGAGGAG-TTG
Int11-*Gh2PS*-R	TGATATGAGAAGCAAACAAGATTTATAGACAGATTAGCTAGGGTTGTTCCGTTAG-CAC
Int20-*Gh2PS*-F	AAGTTTTTTATGAGACGATGATTTCGAAAGGTTCTACAAGGAGATTTCAGAGGAG-TTG
Int20-*Gh2PS*-R	AGCCCTCTTGGAGCTGTGTTATTTACTATTGAGGAAGCTAGGGTTGTTCCGTTAG-CAC
Int33-*Gh2PS*-F	ACCCACATTTTCCTCCTAGCACGTGAATCTTTTAAACAAGGAGATTTCAGAGGAG-TTG
Int33-*Gh2PS*-R	TCTAACTAGTTTGCCGCGACCACCATACAATATTTAGCTAGGGTTGTTCCGTTAG-CAC
Int34-*Gh2PS*-F	GAAGCAAAAATGACTGTAATCCTTATACGGCTGATTCAAGGAGATTTCAGAG-GAGTTG
Int34-*Gh2PS*-R	GTACATTTAATCATGTGTTTTAAAACTGATGATGAAGCTAGGGTTGTTCCGTTAG-CAC
Int35-*CkxPK*-donor	GTCATCTTCCTTGCTTTCGTCTTATTCGATGCTACTAATTGCCTGCTACTCTGGTCCC
Int35-*CkxPK*-donor	ATCTCCACATATCAAGGAATTATCATAACCAGTCTCTTTTCATCTCAC-TTAATCTTCTGTACTCT
Int39-*ScACC1**-F	TGCCCAGCAGGACATTGAAAAAGCAG
Int39-*ScACC1**-R	AGCAGCTGGAGCAGAAGAGGAAG
Int56-*SsXR*-*SsXDH*-F	GAACTTCCATAGGACTTTCTGCA
Int56-*SsXR*-*SsXDH*-R	GAGCTCGTGGTATTCAACCCA
Int1-*SsXKS*-F	GTCCATACTTCTTTTGCAACGTAAATACTAGTCTAAAAGCCAA-TAACTGTCGCCTCTTTTATCTG
Int1-*SsXKS*-R	TCAGGCCTTAAAGGAAATTTGGAACTCCAGTGCCACTTTCGCTCTCAC-TTAATCTTCTGTACTC
Int59-*LmPTA*-donor	TCCAATCAGGGGTTGAGTGA
Int59-*LmPTA*-donor	GGTCAAAGACTTAAGACCGC

Note:

[1] IntX refers to the genomic integration site of *P. pastoris.*

Table S3. List of Gene Coding Sequences Used in This Study.

Gene	Sequences (5′-3′)
Gh2PS	ATGGGAAGTTACTCTAGCGATGATGTGGAGGTTATTCGAGAGGCCGG-<br>TAGAGCCCAGGGCTTAGCGACCATACTTGCGATTGGCACCGCTACTCCACCGAATTCTGT<br>AGCGCAAGCCGACTATGCGGATTACTATTTTCGAGTCACTAAATCAGAGCACATGGTG-<br>GAT-<br>TTAAAGGAGAAATTCAAACGAATTTGTGAAAAGACGGCGATCAAGAAACGGTATCTGGC<br>GTTGACGGAGGACTACCTACAAGAGAATCCAAC-<br>GATGTGCGAATTCATGGCCCCTTCTCTAAACGCTCGCCAGGACCTTGTCGTGACTGGCGT<br>ACCAATGCTCGGTAAAGAGGCAGCCGTAAAGGCCATAGACGAATGGGGATTACCCAA-<br>GAG-<br>TAAAATCACCCACCTAATTTTTGTACAACGGCAGGGGTCGATATGCCGGGGGCAGATTA<br>CCAGCTGGTCAAGCTGCTTGGGTTGAGCCCATCCGTTAAGCGTTACATGTTGTATCAG-<br>CAGGGTTGCGCTGCCGGGGGTACTGTTTTACGCTTGGCCAAAGATCTTGCGGAGAACAA<br>CAAGGGTAGTAGGGTCCTCATCGTGTGCTCCGAAATTACTGCGATACTCTTTCAC-<br>GGACCCAACGAAAATCATCTCGACTCACTTGTCGCTCAAGCCCTGTTCGGTGACGGGGC<br>AGCTGCCCTTATAGTGGGTTCGGGACCGCACCTAGCTGTTGAGCGCCCTATCTTCGA-<br>GATTGTATCAACGGACCAGACAATATTGCCGGATACAGAGAAGGCAATGAAATTACATCT<br>CCGGGAGGGGGGATTAACCTTTCAACTTCATCGGGATGTGCCCCTCATGGTCG-<br>CAAAAAACATCGAAAACGCTGCTGAAAAAGCATTGTCCCCTCTAGGTATTACCGACTGG<br>AATTCTGTGTTTTGGATGGTCCATCCCGGCGGCCGTGCAATACTAGAC-<br>CAAGTAGAAAGGAAACTCAATTTAAAGGAAGACAAGCTTAGGGCGTCGCGTCATGTGCT<br>GTCAGAATATGGGAACCTGATATCGGCGTGTGTACTCTTCATCATCGATGAAGTAC-<br>GGAAAA-<br>GAAGTATGGCTGAAGGAAAGAGCACTACGGGCGAGGGGTTAGACAGCGGCGTTTTGTTT<br>GGATTCGGACCTGGCATGACCGTTGAAACAGTTGTACTAAGGTCGGTTAGAGTGACAG-<br>CAGCTGTAGCAAATGGGAATTGA
*ScACC1**	ATGAGCGAAGAAAGCTTATTCGAGTCTTCTCCACAGAAGATGGAGTAC-<br>GAAATTACAAAC-<br>TACTCAGAAAGACATACAGAACTTCCAGGTCATTTCATTGGCCTCAATACAGTAGATAAA<br>CTAGAGGAGTCCCCGTTAAGGGACTTTGTTAAGAGTCACGGTGGTCACACGGTCATA-<br>TCCAA-<br>GATCCTGATAGCAAATAATGGTATTGCCGCCGTGAAAGAAATTAGATCCGTCAGAAAATG<br>GGCATACGAGACGTTCGGCGATGACAGAAC-<br>CGTCCAATTCGTCGCCATGGCCACCCCAGAA-<br>GATCTGGAGGCCAACGCAGAATATATCCGTATGGCCGATCAATACATTGAAGTGCCAGGT<br>GGTACTAATAATAACAACTACGCTAACGTAGACTTGATCGTAGACATCGCCGAAAGAG-<br>CAGACGTAGACGCCGTATGGGCTGGCTGGGGTCACGCCTCCGAGAATCCACTATTGCCTG<br>AAAAATTGTCCCAGTCTAAGAGGAAAGTCATCTTTATTGGGCCTCCAGGTAAC-<br>GCCATGAGGTCTTTAGGTGATAAAATCTCCTCTACCATTGTCGCTCAAAGTGCTAAAGTCC<br>CATGTATTCCATGGTCTGGTACCGGTGTTGACACCGTTCACGTGGACGAGAAAAC-<br>CGGTCTGGTCTCTGTCGACGATGACATCTATCAAAAGGGTTGTTGTACCTCTCCTGAAGAT<br>GGTTTACAAAAGGCCAAGCGTATTGGTTTTCCTGTCATGAT-<br>TAAGGCATCCGAAGGTGGTGGTGGTAAAGGTATCAGACAAGTTGAACGTGAAGAAGATT<br>TCATCGCTTTATACCACCAGGCAGCCAAC-<br>GAAATTCCAGGCTCCCCCATTTTCATCATGAAGTTGGCCGGTAGAGCGCGTCACTTGGAA<br>GTTCAACTGCTAGCAGATCAGTACGGTACAAATATTTCCTTGTTCGGTAGA-<br>GACTGTTCCGTTCAGAGACGTCATCAAAAAATTATCGAAGAAGCACCAGTTACAATTGCC<br>AAGGCTGAAACATTTCACGAGATGGAAAAGGCTGCCGTCAGACTGGGGAAACTAG-<br>TCGGTTATGTCTCTGCCGGTACCGTGGAGTATCTATATTCTCATGATGATGGAAAATTCTAC<br>TTTTTAGAATTGAACCCAAGATTACAAGTCGAGCATCCAACAAC-<br>GGAAATGGTCTCCGGTGTTAACTTACCTGCAGCTCAATTACAAATCGCTATGGGTATCCCT

	ATGCATAGAATAAGTGACATTAGAACTTTATATGG-<br>TATGAATCCTCATTCTGCCTCAGAAATCGATTTCGAATTCAAAACTCAAGATGCCACCAA<br>GAAACAAAGAAGACCTATTCCAAAGGGTCATTGTACCGCTTGTCGTATCACATCAGAA-<br>GATCCAAACGATGGATTCAAGCCATCGGGTGGTACTTTGCATGAACTAAACTTCCGTTCTT<br>CCTCTAATGTTTGGGGTTACTTCTCCGTGGGTAACAATGGTAATATTCACTCCTTTTCG-<br>GACTCTCAGTTCGGCCATATTTTTGCTTTTGGTGAAAATAGACAAGCTTCCAGGAAACAC<br>ATGGTTGTTGCCCTGAAGGAATTGTCCATTAGGGGTGATTTCAGAACTACTGTGGAA-<br>TACTT-<br>GATCAAACTTTTGGAAACTGAAGATTTCGAGGATAACACTATTACCACCGGTTGGTTGGA<br>CGATTTGATTACTCATAAAATGACCGCTGAAAAGCCTGATCCAACTCTTGCCGTCATTT-<br>GCGGTGCCGCTACAAAGGCTTTCTTAGCATCTGAAGAAGCCCGCCACAAGTATATCGAAT<br>CCTTACAAAAGGGACAAGTTCTATCTAAAGACCTACTGCAAACTATGTTCCCTGTAGAT-<br>TTTATCCATGAGGGTAAAAGATACAAGTTCACCGTAGCTAAATCCGGTAATGACCGTTAC<br>ACATTATTTATCAATGGTTCTAAATGTGATATCAT-<br>ACTGCGTCAACTAGCTGATGGTGGTCTTTTGATTGCCATAGGCGGTAAATCGCATACCATC<br>TATTGGAAAGAAGAAGTTGCTGCTACAAGATTATCCGTTGACTCTATGACTACTTTGTT-<br>GGAAGTTGAAAACGATCCAACCCAGTTGCGTACTCCATCCCCTGGTAAATTGGTTAAATT<br>CTTGGTGGAAAATGGTGAACACATTATCAAGGGCCAACCATATGCAGAAATT-<br>GAAGTTATGAAAATGCAAATGCCTTTGGTTTCTCAAGAAAATGGTATCGTCCAGTTATTAA<br>AGCAACCTGGTTCTACCATTGTTGCAGGTGATATCATGGCTATTATGACTCTTGAC-<br>GATCCATCCAAGGTCAAGCACGCTCTACCATTTGAAGGTATGCTGCCAGATTTTGGTTCTC<br>CAGTTATCGAAGGAACCAAACCTGCCTATAAATTCAAGTCATTAGTGTCTACTTT-<br>GGAAAACATTTTGAAGGGTTATGACAACCAAGTTATTATGAACGCTTCCTTGCAACAATT<br>GATAGAGGTTTTGAGAAATCCAAAACTGCCTTACTCAGAATGGAAAC-<br>TACACATCTCTGCTTTACATTCAAGATTGCCTGCTAAGCTAGATGAACAAATGGAAGAGT<br>TAGTTGCACGTTCTTTGAGACGTGGTGCTGTTTTCCCAGCTAGACAATTAAGTAAATT-<br>GATTGATATGGCCGTGAAGAATCCTGAATACAACCCCGACAAATTGCTGGGCGCCGTCGT<br>GGAACCATTGGCGGATATTGCTCATAAGTACTCTAACGGGTTAGAAGCCCATGAACATTC-<br>TA-<br>TATTTGTCCATTTCTTGGAAGAATATTACGAAGTTGAAAAGTTATTCAATGGTCCAAATGT<br>TCGTGAGGAAAATATCATTCTGAAATTGCGTGATGAAAACCCTAAAGATCTAGA-<br>TAAAGTT-<br>GCGCTAACTGTTTTGTCTCATTCGAAAGTTTCAGCGAAGAATAACCTGATCCTAGCTATCT<br>TGAAACATTATCAACCATTGTGCAAGTTATCTTCTAAAGTTTCTGCCATTTTCTC-<br>TACTCCTCTACAACATATTGTTGAACTAGAATCTAAGGCTACCGCTAAGGTCGCTCTACAA<br>GCAAGAGAAATTTTGATTCAAGGCGCTTTACCTTCGGTCAAGGAAA-<br>GAACTGAACAAATT-<br>GAACATATCTTAAAATCCTCTGTTGTGAAGGTTGCCTATGGCTCATCCAATCCAAAGCGCT<br>CTGAACCAGATTTGAATATCTTGAAGGACTTGATCGATTCTAATTACGTT-<br>GTGTTCGATGTTTTACTTCAATTCCTAACCCATCAAGACCCAGTTGTGACTGCTGCAGCTG<br>CTCAAGTCTATATTCGTCGTGCTTATCGTGCTTACACCATAGGAGATATTAGAGTTCAC-<br>GAAGGTGTCACAGTTCCAATTGTTGAATGGAAATTCCAACTACCTTCAGCTGCGTTCTCC<br>ACCTTTCCAACTGTTAAATCTAAAATGGGTATGAACAGGGCTGTTGCTGTTTCAGATTT-<br>GTCATATGTTGCAAACAGTCAGTCATCTCCGTTAAGAGAAGGTATTTTGATGGCTGTGGAT<br>CATTTAGATGATGTTGATGAAATTTTGTCACAAAGTTTGGAAGTTATTCCTCGTCAC-<br>CAATCTTCTTCTAACGGACCTGCTCCTGATCGTTCTGGTAGCTCCGCATCGTTGAGTAATG<br>TTGCTAATGTTTGTGTTGCTTCTACAGAAGGTTTCGAATCTGAAGAGGAAATTTTGG-<br>TAAGGTTGAGAGAAATTTTGGATTTGAATAAGCAGGAATTAATCAATGCTTCTATCCGTCG<br>TATCACATTTATGTTCGGTTTTAAAGATGGGTCTTATCCAAAGTATTATACTTTTAAC-<br>GGTCCAAATTATAACGAAAATGAAACAATTCGTCACATTGAGCCGGCTTTGGCCTTCCAA<br>CTGGAATTAGGAAGATTGTCCAACTTCAACATTAAACCAATTTTCACTGATAA-<br>TAGAAACATCCATGTCTACGAAGCTGTTAGTAAGACTTCTCCATTGGATAAGAGATTCTTT

	ACAAGAGGTATTATTAGAACGGGTCATATCCGTGATGACATTTCTATTCAAGAA-<br>TATCTGACTTCTGAAGCTAACAGATTGATGAGTGATATATTGGATAATTTAGAAGTCACCG<br>ACACTTCAAATTCTGATTTGAATCATATCTTCATCAACTTCATTGCGGTGTTTGA-<br>TATCTCTCCAGAAGATGTCGAAGCCGCCTTCGGTGGTTTCTTAGAAAGATTTGGTAAGAG<br>ATTGTTGAGATTGCGTGTTTCTTCTGCCGAAATTAGAATCATCATCAAA-<br>GATCCTCAAACAGGTGCCCCAGTACCATTGCGTGCCTTGATCAATAACGTTTCTGGTTATG<br>TTATCAAAACAGAAATGTACACCGAAGTCAAGAACGCAAAAGGTGAATGGG-<br>TATTTAAGTCTTTGGGTAAACCTGGATCCATGCATTTAAGACCTATTGCTACTCCTTACCCT<br>GTTAAGGAATGGTTGCAACCAAAACGTTATAAGGCACACTTGATGGGTACCACAT-<br>ATGTC-<br>TATGACTTCCCAGAATTATTCCGCCAAGCATCGTCATCCCAATGGAAAAATTTCTCTGCAG<br>ATGTTAAGTTAACAGATGATTTCTTTATTTCCAACGAGTTGATTGAAGATGAAAAC-<br>GGCGAATTAACTGAGGTGGAAAGAGAACCTGGTGCCAACGCTATTGGTATGGTTGCCTTT<br>AAGATTACTGTAAAGACTCCTGAATATCCAAGAGGCCGTCAATTTGTTGTTGTTGCTAAC-<br>GA-<br>TATCACATTCAAGATCGGTTCCTTTGGTCCACAAGAAGACGAATTCTTCAATAAGGTTACT<br>GAATATGCTAGAAAGCGTGGTATCCCAAGAATTTACTTGGCTG-<br>CAAACTCAGGTGCCAGAATTGGTATGGCTGAAGAGATTGTTCCACTATTTCAAGTTGCAT<br>GGAATGATGCTGCCAATCCGGACAAGGGCTTCCAA-<br>TACTTATACTTAACAAGTGAAGGTATGGAAACTTTAAAGAAATTTGACAAAGAAAATTCT<br>GTTCTCACTGAACGTACTGTTATAAACGGTGAAGAAAGATTTGTCATCAAGA-<br>CAATTATTGGTTCTGAAGATGGGTTAGGTGTCGAATGTCTACGTGGATCTGGTTTAATTGCT<br>GGTGCAACGTCAAGGGCTTACCACGATATCTTCACTATCACCTTAGTCACTT-<br>GTAGATCCGTCGGTATCGGTGCTTATTTGGTTCGTTTGGGTCAAAGAGCTATTCAGGTCGA<br>AGGCCAGCCAATTATTTTAACTGGTGCTCCTGCAATCAACAAAATGCTGGGTAGA-<br>GAAGTTTATACTTCTAACTTACAATTGGGTGGTACTCAAATCATGTATAACAACGGTGTTT<br>CACATTTGACTGCTGTTGACGATTTAGCTGGTGTAGAGAAGATTGTT-<br>GAATGGATGTCTTATGTTCCAGCCAAGCGTAATATGCCAGTTCCTATCTTGGAAACTAAAG<br>ACACATGGGATAGACCAGTTGATTTCACTCCAACTAATGATGAAACTTACGATGTAA-<br>GATGGATGATTGAAGGTCGTGAGACTGAAAGTGGATTTGAATATGGTTTGTTTGATAAAG<br>GGTCTTTCTTTGAAACTTTGTCAGGATGGGCCAAAGGTGTTGTCGTTGG-<br>TAGAGCCCGTCTT-<br>GGTGGTATTCCACTGGGTGTTATTGGTGTTGAAACAAGAACTGTCGAGAACTTGATTCCT<br>GCTGATCCAGCTAATCCAAATAGTGCTGAAACATTAATTCAAGAACCTGGTCAAGTTTGG-<br>CATCCAAACTCCGCCTTCAAGACTGCTCAAGCTATCAATGACTTTAACAACGGTGAACAA<br>TTGCCAATGATGATTTTGGCCAACTGGAGAGGTTTCTCTGGTGGTCAACGTGA-<br>TATGTTCAACGAAGTCTTGAAGTATGGTTCGTTTATTGTTGACGCATTGGTGGATTACAAA<br>CAACCAATTATTATCTATATCCCACCTACCGGTGAACTAAGAGGTGGTTCATGGGTTGTT-<br>GTCGATCCAACTATCAACGCTGACCAAATGGAAATGTATGCCGACGTCAACGCTAGAGCT<br>GGTGTTTTGGAACCACAAGGTATGGTTGGTATCAAGTTCCGTAGAGAAAAATTGCTG-<br>GACAC-<br>CATGAACAGATTGGATGACAAGTACAGAGAATTGAGATCTCAATTATCCAACAAGAGTTT<br>GGCTCCAGAAGTACATCAGCAAATATCCAAGCAATTAGCTGATCGTGAGAGAGAAC-<br>TATTGCCAATTTACGGACAAATCAGTCTTCAATTTGCTGATTTGCACGATAGGTCTTCACG<br>TATGGTGGCCAAGGGTGTTATTTCTAAGGAACTGGAATGGACCGAGGCAC-<br>GTCGTTTCTTCTTCTGGAGATTGAGAAGAAGATTGAACGAAGAATATTTGATTAAAAGGT<br>TGAGCCATCAGGTAGGCGAAGCATCAAGATTAGAAAAGATCGCAAGAATT-<br>AGATCGTGG-<br>TACCCTGCTTCAGTGGACCATGAAGATGATAGGCAAGTCGCAACATGGATTGAAGAAAA<br>CTACAAAACTTTGGACGATAAACTAAAGGGTTTGAAATTAGAGTCATTCGCTCAA-<br>GACTTAGCTAAAAAGATCAGAAGCGACCATGACAATGCTATTGATGGATTATCTGAAGTT<br>ATCAAGATGTTATCTACCGATGATAAAGAAAAATTGTTGAAGACTTTGAAATGA

CkxPK	ATGGCTGATTTCGACTCAAAGGAATACTTAGAATTGGTTGATAAATGGTGGAGAGCTACT-<br>A-<br>ACTACTTGTCCGCTGGTATGATCTTCTTGAAGTCAAACCCATTATTTTCCGTTACTAACACA<br>CCTATCAAGGCTGAAGATGTTAAAGTCAAGCCAATTGGTCATTGGGGTACTA-<br>TATCTGGTCAAACATTCTTGTATGCTCACGCAAACAGATTGATTAACAAATACGGTTTGAA<br>TATGTTTTACGTTGGTGGTCCAGGTCATGGTGGTCAAGTAATGGTTACAAACGCTTACTT-<br>GGATGGTGCATATACCGAAGACTACCCTGAAATTACTCAAGATATCGAGGGTATGAGTCA<br>TTTGTTTAAAAGATTCTCTTTCCCAGGTGGTATTGGTTCACATATGACTGCTCAAACAC-<br>CTGGTTCATTGCACGAAGGTGGTGAATTGGGTTATTCCTTAAGTCATGCCTTCGGTGCTGT<br>TTTAGATAATCCAGACCAAGTCGCCTTTGCTGTTGTCGGTGACGGTGAA-<br>GCAGAAACAGGTCCTTCTATGGCCTCATGGCACTCCATAAAATTTTTGAATGCCAAGAAC<br>GATGGTGCTGTTTTACCAGTCTT-<br>GGACTTAAATGGTTTCAAAATCTCTAACCCTACAATTTTTAGTAGAATGTCTGATGAAGAA<br>ATAACCAAGTTTTTCGAAGGTTTGGGTTACTCACCAAGATTCATTGAAAAC-<br>GATGACATCCATGATTATGCAACTTACCACCAATTGGCTGCAAACATCTTGGATCAAGCT<br>ATCGAAGACATCCAAGCAATCCAAAATGATGCCAGAGAAAACGGTAAATACCAAGAC-<br>GGTGAAATACCAGCCTGGCCTGTTATTATAGCTAGATTGCCAAAGGGTTGGGGTGGTCCT<br>ACACATGATGCATCCAATAACCCAATCGAAAATAGTTTTAGAGCCCATCAAGTTCCATT-<br>GCCTTTAGAACAACACGATTTGGCAACTTTACCAGAATTCGAAGACTGGATGAACTCTTA<br>CAAGCCTGAAGAATTGTTTAATGCTGATGGTTCATTGAAAGAC-<br>GAATTAAAGGCAATCGCCCCAAAAGGTGACAAGAGAATGTCCGCCAATCCTATTACAAA<br>CGGTGGTGCTGATAGAAGTGACTTGAAGTTACCAAACTGGAGAGAATTCGCAAACGATA-<br>TAAACGATGACACTAGAGGTAAAGAATTCGCTGATTCAAAGAGAAACATGGACATGGCA<br>ACATTGTCTAACTACTTAGGTGCCGTTTCACAATTGAATCCAACCAGATTCAGAT-<br>TTTTCGGTCCTGATGAAACTATGTCCAATAGATTGTGGGGTTTGTTTAATGTCACCCCTAG<br>ACAATGGATGGAAGAAATTAAAGAACCACAAGATCAATTGTTGTCTCCTACTGG-<br>TAGAATCATTGACTCACAATTATCCGAACATCAAGCAGAAGGTTGGTTGGAAGGTTATAC<br>CTTAACTGGTAGAGTCGGTATTTTCGCTAGTTACGAATCATTTTTGAGAGTAGTTGATAC-<br>CATGGTAACTCAACATTTCAAGTGGTTGAGACACGCTTCTGAACAAGCATGGAGAAACG<br>ATTACCCATCCTTGAACTTAATAGCCACAAGTACCGCTTTCCAACAA-<br>GATCATAATGGTTACACACACCAAGACCCAGGCATGTTGACCCATTTGGCAGAAAAGAA<br>ATCTAACTTCATCAGAGAATATTTGCCTGCTGATGGTAACTCTTTGTTAGCTGTACAA-<br>GAAA-<br>GAGCATTTTCAGAAAGACATAAGGTTAATTTGTTGATCGCTTCTAAGCAACCTAGACAAC<br>AATGGTTCACTGTAGAAGAAGCAGAAGTTTTGGCCAACGAAGGTTTAAAAA-<br>TAATCGATTGGGCATCTACTGCCCCATCTTCAGATGTTGACATTACATTTGCTTCAGCAGG<br>TACAGAACCTACCATAGAAACTTTGGCCGCTTTGTGGTTAATCAATCAA-<br>GCATTTCCAGATGTCAAGTTTAGATACGTAAACGTCGTAGAATTGTTGAGATTGCAAAAG<br>AAATCTGAACCTAACATGAACGATGAAAGAGAATTAAGTGCTGAAGAATTCAA-<br>TAAGTACTTCCAAGCAGACACACCAGTTATTTTCGGTTTTCATGCTTACGAAAACTTAATC<br>GAATCATTTTTCTTTGAAAGAAAGTTTACTGGTGACGTCTATGTACACGGTTACAGAGAA-<br>GATGGTGACATTACTACAACCTATGATATGAGAGTTTACTCCCATTTGGACAGATTCCACC<br>AAGCCAAAGAAGCAGCCGAAATCTTGAGTGCTAACGGTAAAATAGATCAAGCTG-<br>CAGCCGACACTTTCATAGCTAAAATGGATGACACATTGGCAAAGCATTTTCAAGTTACCA<br>GAAATGAAGGTAGAGATATCGAAGAATTCACTGATTGGACCTGGTCACCATTAAAATAA-<br>TAA
LmPTA	ATGAAGCTAATGGAAAACATCTTCGGGCTGGCCAAGGCGGACAAAAAAAA-<br>GATCGTGTT-<br>GGCGGAAGGCGAAGAGGAGCGTAACATTAGGGCTTCCGAGGAAATAATCAGAGACGGG<br>ATAGCCGATATAATCCTAGTTGGCTCAGAATCAGTCATTAAGGAAAATGCCGCTAAATTT-<br>GGCGTGAATCTGGCAGGGGTCGAGATAGTCGATCCAGAAACGTCCTCCAAGACTGCCGG<br>CTACGCGAACGCTTTCTATGAGATCCGTAAAAATAAGGGTGTCACACTAGAAAAAGCG-

	GA-<br>TAAAATAGTCCGTGATCCAATTTATTTTGCCACCATGATGGTCAAACTAGGTGATGCTGAT<br>GGGCTAGTGTCTGGAGCCATTCACACAACGGGTGATTTACTAAGGCCAGGCCTG-<br>CAGATCGTTAAGACAGTTCCCGGCGCGAGTGTGGTGTCCTCTGTCTTCTTGATGAGTGTTC<br>CTGACTGCGAGTACGGCGAG-<br>GATGGTTTTCTGTTATTCGCTGACTGTGCTGTTAATGTATGCCCGACCGCAGAGGAATTATC<br>TAGCATAGCAATCACAACCGCCGAGACGGCAAAAAACCTTTGTAA-<br>GATCGAGCCCAGAGTCG-<br>CAATGCTATCTTTCTCTACAATGGGTTCCGCGTCCCATGAACTAGTCGACAAAGTGACTAA<br>GGCCACAAAGTTGGCTAAGGAGGCACGTCCCGACTTGGATATCGACGGTGAATT-<br>GCAATT-<br>AGATGCGTCACTAGTAAAGAAAGTAGCTGACCTAAAGGCTCCCGGCTCCAAGGTGGCAG<br>GTAAGGCTAATGTGTTGATTTTTCCGGATATACAGGCTGGCAACATAGGGTATAAACTAG-<br>TCCAGCGTTTTGCGAAAGCTGAAGCAATCGGACCCATTTGCCAAGGATTTGCCAAGCCTA<br>TAAACGATTTGAGTAGAGGATGTTCTGTCGACGACATTGTAAAGGTCGTGGCGGTCAC-<br>GGCGGTTCAGGCGCAAGCGCAAGGCTAATAA
SsXR	ATGCCTTCTATTAAGTTGAACTCTGGTTACGACATGCCAGCCGTCGGTTTCGGCTGTT-<br>GGAAAGTCGACGTCGACACCTGTTCTGAACAGATCTACCGTGCTATCAAGACCGGTTAC<br>AGATTGTTCGACGGTGCCGAAGATTACGCCAACGAAAAGTTAGTTGGTGCCGGTGTCAA-<br>GAAGGCCATTGACGAAGGTATCGTCAAGCGTGAAGACTTGTTCCTTACCTCCAAGTTGTG<br>GAACAACTACCACCACCCAGACAACGTCGAAAAGGCCTT-<br>GAACAGAACCCTTTCTGACTT-<br>GCAAGTTGACTACGTTGACTTGTTCTTGATCCACTTCCCAGTCACCTTCAAGTTCGTTCCA<br>TTAGAAGAAAAGTACCCACCAGGATTCTACTGTGGTAAGGGTGACAACTTCGACTAC-<br>GAA-<br>GATGTTCCAATTTTAGAGACCTGGAAGGCTCTTGAAAAGTTGGTCAAGGCCGGTAAGATC<br>AGATCTATCGGTGTTTCTAACTTCCCAGGTGCTTTGCTCTTGGACTTGTTGAGAGGTGC-<br>TAC-<br>CATCAAGCCATCTGTCTTGCAAGTTGAACACCACCCATACTTGCAACAACCAAGATTGAT<br>CGAATTCGCTCAATCCCGTGGTATTGCTGTCAC-<br>CGCTTACTCTTCGTTCGGTCCTCAATCTTTCGTTGAATTGAACCAAGGTAGAGCTTTGAAC<br>ACTTCTCCATTGTTCGAGAACGAAACTATCAAGGCTATCGCTGCTAAGCACGG-<br>TAAGTCTCCAGCTCAAGTCTTGTTGAGATGGTCTTCCCAAAGAGGCATTGCCATCATTCC<br>AAAGTCCAACACTGTCCCAAGATTGTTGGAAAACAAGGACGTCAACAGCTTCGACTT-<br>GGAC-<br>GAACAAGATTTCGCTGACATTGCCAAGTTGGACATCAACTTGAGATTCAACGACCCATGG<br>GACTGGGACAAGATTCCTATCTTCGTCTAA
SsXDH	ATGACTGCTAACCCTTCCTTGGTGTTGAACAAGATCGACGACATTTCGTTCGAAACTTAC-<br>GATGCCCCAGAAATCTCTGAACCTACCGATGTCCTCGTCCAGGTCAAGAAAACCGGTATC<br>TGTGGTTCCGACATCCACTTCTACGCCCATGGTAGAATCGGTAACTTCGTTTTGACCAA-<br>GCCAATGGTCTTGGGTCACGAATCCGCCGGTACTGTTGTCCAGGTTGGTAAGGGTGTCAC<br>CTCTCTTAAGGTTGGTGACAACGTCGCTATCGAACCAGGTATTCCATCCAGAT-<br>TCTCCGAC-<br>GAATACAAGAGCGGTCACTACAACTTGTGTCCTCACATGGCCTTCGCCGCTACTCCTAAC<br>TCCAAGGAAGGCGAACCAAACCCACCAGGTAC-<br>CTTATGTAAGTACTTCAAGTCGCCAGAA-<br>GACTTCTTGGTCAAGTTGCCAGACCACGTCAGCTTGGAACTCGGTGCTCTTGTTGAGCCA<br>TTGTCTGTTGGTGTCCACGCCTCCAAGTTGGGTTCCGTTGCTTTCGGCGACTACGTT-<br>GCCGTCTTTGGTGCTGGTCCTGTTGGTCTTTTGGCTGCTGCTGTCGCCAAGACCTTCGGTG<br>CTAAGGGTGTCATCGTCGTTGACATTTTCGACAACAAGTTGAAGATGGCCAAGGACATT-<br>GGTGCTGCTACTCACACCTTCAACTCCAAGACCGGTGGTTCTGAAGAATTGATCAAGGCT<br>TTCGGTGGTAACGTGCCAAACGTCGTTTTGGAATGTACTGGTGCTGAACCTT-

	GTATCAAGTT-<br>GGGTGTTGACGCCATTGCCCCAGGTGGTCGTTTCGTTCAAGTTGGTAACGCTGCTGGTCC<br>AGTCAGCTTCCCAATCACCGTTTTCGCCATGAAGGAATTGACTTT-<br>GTTCGGTTCTTTCAGA-<br>TACGGATTCAACGACTACAAGACTGCTGTTGGAATCTTTGACACTAACTACCAAAACGGT<br>AGAGAAAATGCTCCAATTGACTTTGAACAATTGATCACCCACAGATA-<br>CAAGTTCAAGGACGC-<br>TATTGAAGCCTACGACTTGGTCAGAGCCGGTAAGGGTGCTGTCAAGTGTCTCATTGACGG<br>CCCTGAGTAA
SsXKS	ATGACCACTACCCCATTTGATGCTCCAGATAA-<br>GCTCTTCCTCGGGTTCGATCTTTCGACTCAGCAGTTGAAGATCATCGTCACCGATGAAAA<br>CCTCGCTGCTCTCAAAACCTACAATGTCGAGTTCGA-<br>TAGCATCAACAGCTCTGTCCAGAAGGGTGTCATTGCTATCAACGACGAAATCAGCAAGG<br>GTGCCATTATTTCCCCCGTTTACATGTGGTTGGATGCCCTTGACCATGTTTTTGAAGA-<br>CATGAAGAAGGACGGATTCCCCTTCAACAAGGTTGTTGGTATTTCCGGTTCTTGTCAACA<br>GCACGGTTCGGTATACTGGTCTAGAACGGCCGAGAAGGTCTTGTCCGAATTGGAC-<br>GCTGAATCTTCGTTATCGAGCCAGATGAGATCTGCTTTCACCTTCAAGCACGCTCCAAAC<br>TGGCAGGATCACTCTACCGGTAAAGAGCTTGAAGAGTTCGAAAGAG-<br>TGATTGGTGCTGATGCCTTGGCTGATATCTCTGGTTCCAGAGCCCATTACAGATTCACAGG<br>GCTCCAGATTAGAAAGTTGTCTACCAGATTCAA-<br>GCCCGAAAAGTACAACAGAACTGCTCG-<br>TATCTCTTTAGTTTCGTCATTTGTTGCCAGTGTGTTGCTTGGTAGAATCACCTCCATTGAAG<br>AAGCCGATGCTTGTGGAATGAACTTGTACGATATCGAAAAGCGCGAGTTCAACGAA-<br>GAGCTCTTGGCCATCGCTGCTGGTGTCCACCCTGAGTTGGATGGTGTAGAACAAGACGGT<br>GAAATTTACAGAGCTGGTATCAATGAGTTGAAGAGAAAGTTGGGTCCTGTCAAAC-<br>CTATAACATACGAAAGCGAAGGTGACATTGCCTCTTACTTTGTCACCAGATACGGCTTCA<br>ACCCCGACTGTAAAATCTACTCGTTCACCGGAGACAATTTGGCCACGATTATCTCGTT-<br>GCCTTTGGCTCCAAATGATGCTTTGATCTCATTGGGTACTTCTACTACAGTTTTAATTATCA<br>CCAAGAACTACGCTCCTTCTTCTCAATACCATTTGTTTAAACATCCAACCATGCCTGAC-<br>CAC-<br>TACATGGGCATGATCTGCTACTGTAACGGTTCCTTGGCCAGAGAAAAGGTTAGAGACGA<br>AGTCAACGAAAAGTTCAATGTAGAAGACAAGAAGTCGTGG-<br>GACAAGTTCAATGAAATCTT-<br>GGACAAATCCACAGACTTCAACAACAAGTTGGGTATTTACTTCCCACTTGGCGAAATTGT<br>CCCTAATGCCGCTGCTCAGATCAAGAGATCGGTGTTGAACAGCAAGAACGAAATT-<br>GTAGAC-<br>GTTGAGTTGGGCGACAAGAACTGGCAACCTGAAGATGATGTTTCTTCAATTGTAGAATCA<br>CAGACTTTGTCTTGTAGATTGAGAACTGGTCCAATGTTGAGCAAGAGTGGAGAT-<br>TCTTCTGCTTCCAGCTCTGCCTCACCTCAACCAGAAGGTGATGGTACAGATTTGCACAAG<br>GTCTACCAAGACTTGGTTAAAAAGTTTGGTGACTTGTTCACTGATGGAAAGAAGCAAAC-<br>CTTTGAGTCTTTGACCGCCAGACCTAACCGTTGTTACTACGTCGGTGGTGCTTCCAACAA<br>CGGCAGCATTATCCSCAAGATGGGTTCCATCTTGGCTCCCGTCAACGGAAAC-<br>TACAAGGTT-<br>GACATTCCTAACGCCTGTGCATTGGGTGGTGCTTACAAGGCCAGTTGGAGTTACGAGTGT<br>GAAGCCAAGAAGGAATGGATCGGATACGATCAGTATATCAACAGATTGTTT-<br>GAAGTAAGTGACGAGATGAATCTGTTCGAAGTCAAGGATAAATGGCTCGAATATGCCAA<br>CGGGGTTGGAATGTTGGCCAAGATGGAAAGTGAATTGAAACACTAA

Table S4. Comparison of TAL Titer, Yield, Productivity from different carbon sources.

Strain	Medium	Titer (g/L)	Yield (g/g)	Volumetric productivity (g/L·h)
PpTAL1	YPD	0.95	0.0477	0.0133
PpTAL2	YPD	0.93	0.0467	0.0130
PpTAL3	YPD	1.07	0.0536	0.0149
PpTAL4	YPD	1.51	0.0753	0.0209
PpTAL5	YPD	2.01	0.100	0.0279
PpTAL6	YPD	2.44	0.122	0.0339
PpTAL7	YPD	2.55	0.127	0.0354
PpTAL8	YPD	2.72	0.136	0.0378
PpTAL10	SCD	0.50	0.0251	0.00696
PpTAL11	SCD	0.53	0.0266	0.00739
PpTAL10	SCX	0.54	0.0271	0.00754
PpTAL11	SCX	0.83	0.0413	0.0115
PpTAL8	SCM	0.021	0.00103	0.000285
PpTAL9	SCM	0.057	0.00285	0.000793
